# Optimizing the management of advanced non-small-cell lung cancer: a personal view

**DOI:** 10.3747/co.v16i4.465

**Published:** 2009-08

**Authors:** M.D. Vincent

**Affiliations:** * Medical Oncology, London Regional Cancer Program, London, ON

**Keywords:** Advanced non-small-cell lung cancer, treatment, personal view

## Abstract

The management of advanced non-small-cell lung cancer (a-nsclc) is currently undergoing one of its rare paradigm shifts. Just as the nihilism of the 1970s gave way to the empiricism of the 1980s and 1990s, so the current decade has seen the first truly rational therapies based on informed design. In addition, molecular markers and traditional parameters can now be combined to provide a framework of knowledge that will guide the application of not just the new therapies, but also the older ones that remain effective. This framework—as important a component of the rational paradigm as the new drugs themselves are—is necessary to decide who should and, crucially, who should not receive the various components of this rapidly expanding armamentarium. Here, I have provided a historical overview of the drug treatment of a-nsclc, a mini-review of important new data, and an integrative approach that tries to ensure that patients receive the optimal treatment choice at the appropriate time.

The speed at which new knowledge now arrives, coupled with the persistent high level of unmet medical need, suggests that the traditional pace of evidence-based review needs to be accelerated. Indeed, the increased scope for personalized management constitutes something of a challenge to “business as usual” evidence-based medicine. As a result, substantial investment on the part of payers, which may or may not be possible, will be required. In the meantime, some patients may wish and may be financially able to take advantage of modern developments before they have been fully digested by the public-payer system. Responsive clinicians face difficult tradeoffs as they try to balance the pros and cons of early adoption versus excessive conservatism.

The present article is my personal view of how to navigate these waters, and although it is written especially for patients who like to be the captain of their own ship, there is good reason to believe that all patients will eventually be managed by similar, if not identical, means. Nonetheless, the recommendations herein should not be construed as appropriately reviewed provincial or national guidelines. Finally, if appropriate, a clinical trial should always be offered.

## 1. INTRODUCTION

Non-small-cell lung cancer (nsclc) is a heterogeneous collection of entities sharing only three properties: they arise in the post-tracheal respiratory tree, they are carcinomas, and they are not, morphologically, small-cell. This low barrier to entry is reflected in the World Health Organization 2004 classification 1, which lists 44 subtypes. At least some of these histotypes are surrogates for a suite of molecular characteristics that both constrain and enable the activities of various drugs, classical and new alike 2–8. These new discoveries regarding targets and targeted agents can now be combined with traditional parameters—for example, histology and smoking status—and with the older drugs into a kind of “modern synthesis,” which will allow for a welcome and overdue level of sophistication in management.

Nonetheless, there is also a danger of multiplying complexity beyond what is necessary: “that which can be explained with less is explained in vain with more.” Unfortunately, novel biomarker distribution does not always fall neatly within classical histologic subtypes, and practitioners should be prepared for subgroups to merge and to fragment in novel ways. This novelty will be reflected in the algorithm presented later, particularly for adenocarcinoma. Note that the management of elderly or unfit patients is not covered. It must be emphasized that considerable uncertainty remains in some of the recommendations.

## 2. SOURCES OF HETEROGENEITY

### 2.1 Goals of Management

In advanced nsclc (a-nsclc), the most important goals are to prolong survival and to relieve or prevent symptoms. Efforts to minimize toxicity are also critical and should focus on avoidance of unnecessary toxicity and especially of intolerable toxicity. Some novel treatments are considerably less toxic than are older drugs; others are at least as problematic, although in new and challenging ways.

### 2.2 Principles of Management: What Has Changed?

The traditional priorities—to provide appropriate and realistic information, and to seek and satisfy the patient’s wishes—are unchanged. The next task is to obtain rapid control of aggressive, symptomatic disease; to select the correct modality (including radiation, if appropriate) and regimen; and to administer treatment sooner rather than later. The potential for harm, and strategies for avoidance, should be considered. What is new is the need to appreciate that most patients will receive serial treatments, each potentially constraining the choice of what follows. The entire course therefore needs to be mapped out, at least in a flexible way, at the beginning. An attempt should be made to stay within a standard-of-care framework. If emerging data make this attempt difficult, new options should be debated with the patient and with colleagues, guided by the patient’s ability to access the drug or drugs.

### 2.3 Sources of Uncertainty

In the absence of adequate biopsy tissue, making rational decisions becomes increasingly difficult. Unlike many other cancers, nsclc often presents in an advanced form that is difficult to biopsy properly or that results in a biopsy reflective of a diagnostic culture of minimalism. Serum studies of tumour-derived markers may be a way forward, but experience to date is not encouraging 9.

Lack of adequate tissue apart, the full implications of many parameters have yet to emerge—for example, *KRAS* mutation status. A transitional phase in which decisions will frequently have to be made under uncertainty is inevitable. Guidelines, while important, cannot resolve all dilemmas. Likewise, new knowledge can both raise and lower the level of uncertainty. How to cope is the hallmark of the sophisticated clinician. Rigid and simplistic guidelines, a requirement for absolute proof, and a dogmatic adherence to old data are how not to proceed, but unnecessary gambling is a similarly poor choice.

### 2.4 Primum Non Nocere

The ancient precept *primum non nocere* has always posed a challenge for medical oncologists. It is particularly important to appreciate that there is a taxonomy of harm, because subtle forms of harm exist and can, with skill, be avoided.

Harm can be active or passive. Passive harm arises when, for example, a decision is made to avoid treatment and just to observe the patient when active intervention is more appropriate. One instance might be a decision to forgo maintenance treatment, which has now been shown to improve progression-free survival (pfs) and the disease control rate, and even to prolong survival, as discussed later in this article. Another instance of passive harm would be selection of an inferior regimen when a superior regimen is available—for example, the continued use of docetaxel in patients with non-squamous histology in the second line, when a better and less toxic alternative, pemetrexed, is available.

Active harm is usually understood to be the creation of toxicity, although this conception may be too limited. Concerning toxicity, however, it is useful to consider what the patient wishes to avoid, to what the patient is vulnerable, and the consequences that would ensue should the worst actually happen. The classical and novel regimens all have well-described and somewhat variable propensities to cause a range of toxicities, a situation that allows for considerable personalization of treatment (Table I). A free-access Web site (www.predictpatientevents.com) that individualizes quantitative risk determination for several toxicities has been developed, with more risk models in preparation.

Yet recent evidence indicates that another form of active harm might be possible: that of direct or indirect tumour acceleration. Small-molecule epidermal growth factor receptor (egfr) tyrosine kinase inhibitors (tkis) have, in at least three trials, clearly or probably exhibited this effect. In tribute (Tarceva Responses in conjunction with Paclitaxel and Carboplatin), a failed study of chemotherapy with and without erlotinib, patients were analyzed by *KRAS* status; *KRAS-*mutant patients on chemotherapy with erlotinib had a statistically worse survival than did *KRAS-*mutant patients on chemotherapy with placebo 16. In br.21, which tested erlotinib against placebo in the second and third lines, *KRAS-*mutant patients also had worse survival [hazard ratio (hr): 1.67], but given the small numbers analyzed, the *p* value was not significant 4. Finally, the Southwest Oncology Group study 0023, which tested the addition of gefitinib (against placebo) in stage iii patients as maintenance post chemoradiation, had to be prematurely stopped because the gefitinib arm experienced a marked and statistically worse survival 17, with the excess deaths being attributable to cancer.

The foregoing findings remain unexplained, but they cannot easily be dismissed. *KRAS* mutations are definitely, or probably, involved 18. Caution should be exercised if these drugs are being considered in ex- or current smokers with adenocarcinoma, about 40% of whom harbour *ras-*mutated tumours 19. If a *ras* mutation is known to be present, egfr-tkis should be avoided or used as a last resort.

### 2.5 The Status Quo Ante

In 1995, a meta-analysis was published establishing the benefit of cisplatin-based “second-generation” regimens over best supportive care 20. A variety of such regimens—for example, cisplatin–etoposide, mitomycin–vinblastine–cisplatin—was actively explored by the Eastern Cooperative Oncology group (ecog) 21, with cisplatin–etoposide exhibiting the highest proportion of 1-year survivors (25%). When tested later against cisplatin–paclitaxel, cisplatin–etoposide was only marginally inferior, and then only because of the inclusion of stage iiib patients 22. In another study, carboplatin–paclitaxel was slightly inferior to cisplatin–etoposide in respect of survival, but was felt to result in superior quality of life 23. These borderline results nonetheless ushered in the era of the third-generation platinum doublets, combining either cisplatin or carboplatin with one of gemcitabine, vinorelbine, paclitaxel, or docetaxel.

These third-generation regimens, which until very recently were the standard of care in the first line, exhibit only modest efficacy differences 11,24,25. Meta-analyses have shown that chemotherapy is better if platinum-based 26; that either gemcitabine-containing 27 or docetaxel-containing 28 doublets are superior; that doublets are better than a single agent, but that triplet cytotoxic regimens are not superior to a platinum doublet 29; and that cisplatin-based doublets are superior to carboplatin-based doublets 30. Furthermore, prolonging treatment beyond 4–6 cycles, whether with the same or different drugs, appears not to prolong survival 31 despite hints that pfs or symptom control might be improved 32,33.

The value of second-line chemotherapy was demonstrated when docetaxel proved superior to placebo in respect of survival and symptom control 34. Pemetrexed was then compared with docetaxel in that setting, with virtually identical objective response and survival. Pemetrexed, however, was considerably less toxic than docetaxel, and was therefore widely approved for that indication 35. A subsequent, post-hoc analysis 36 showed that pemetrexed was superior in pfs (hr: 0.82; *p* = 0.076) and overall survival [os (hr: 0.78; *p* = 0.047)] to docetaxel in patients with adenocarcinoma or large-cell (“non-squamous”) disease, whereas the reverse was true in the squamous carcinoma subgroup (pfs hr: 1.40; *p* = 0.046; os hr: 0.78; *p* = 0.047). This unexpected finding led to a more restricted approval for pemetrexed in the second line in some jurisdictions.

It should be noted that the survival hr advantage for large-cell disease (0.27) was more impressive than that for adenocarcinoma (0.92) 37. Nonetheless, the response rate and pfs were both in favour of pemetrexed in adenocarcinoma, and these results, together with lower toxicity and a shorter infusion time, drove the replacement of docetaxel by pemetrexed in all non-squamous second-line patients. Pemetrexed seems to be unusual in this selectivity by histology, and in this respect, it might be considered a “fourth-generation” type of cytotoxic, in that it is targeted in a manner that is clinically exploitable: that is, targeted clinically in addition to having a known molecular target. Other cytotoxics (first-, second-, and third-generation) are not known to be histologically targeted except in the sense that they are usually more active in small-cell lung cancer than in nsclc.

A further publication on the pemetrexed work 38 showed that the drug’s superior tolerability profile was particularly important in elderly patients regardless of histology, although younger patients also enjoyed considerably less toxicity on pemetrexed. Yet another publication from the same study indicated that, in the whole patient population, symptom relief was correlated with objective response status and pfs 39. That analysis implies an importance for treating symptomatic patients with drugs to which they are most likely to respond.

Other studies have examined the role of the egfr-tkis in the second (or third) line. The br.21 40 study compared erlotinib with placebo in this setting and showed a significant benefit in survival and symptom control for erlotinib, at the cost of skin rash and some diarrhea. This result led to the approval of erlotinib. It has been claimed that erlotinib benefited all subgroups of patients, although certain molecular parameters and clinical features seemed to indicate considerable selectivity 4,41. Erlotinib is well recognized as a targeted type of therapy (in the sense of having a known molecular target, a driver of the malignant phenotype); however, whether erlotinib targeted particular subgroups of patients—and if so, to what extent—was unclear. In particular, it was not clear whether the types of patients who would not benefit, and who therefore should not in future be offered erlotinib, were identifiable.

Careful scrutiny of the br.21 subgroup data as published reveals that non-smokers exhibited better survival improvement than did smokers (hr: 0.42, *p* < 0.001 compared with hr: 0.86, *p* = 0.141; interaction *p* = 0.0109). Among the smokers, however, the squamous patients enjoyed an excellent benefit (hr: 0.66; *p* = 0.009). This finding raised the question of how well the residual smokers could have done once the squamous patients had been extracted. A subsequent publication 42 revealed that the hr for ex- or current smokers with adenocarcinoma was 0.94 ( *p* = 0.686) and that median survival actually favoured placebo (5.22 months vs. 4.83 months).

Note, then, the heterogeneity, especially within adenocarcinoma: the never-smokers who do well on egfr-tkis, and the ex- or current smokers who, overall, do not do so well. This observation raises the question of whether it is useful to consider adenocarcinoma a single category. Clearly, from the viewpoints of causation, prognosis, and egfr-tki therapy, it is not; but the present article later shows that, from the viewpoint of relative benefit on pemetrexed, it is.

Gefitinib, also an egfr-tki, was approved in Canada for use after chemotherapy failure, but it was withdrawn when the large isel (Iressa Survival Evaluation in Lung Cancer) trial (placebo comparison) did not show benefit outside of Asia 43. The study patients were all strictly chemotherapy-refractory, a situation different from that in br.21, in which only 28% of patients were refractory. Subsequently, gefitinib showed non-inferiority to docetaxel in interest (Iressa Non-small Cell Lung Cancer Trial Evaluating Response and Survival Against Taxotere) 44 in the second line, and superiority in pfs or objective response rate (orr) over carboplatin–paclitaxel in Asian patients in the first line (providing that *EGFR* is mutated) in ipass (Iressa Pan Asia Study) 45. However, in wild-type *EGFR* patients, gefitinib was markedly inferior to chemotherapy in respect of orr and pfs, and possibly in respect of survival. The strong indication was that gefitinib should not be given to wild-type *EGFR* never-smokers, and that if a patient’s *EGFR* status is unknown, induction chemotherapy (followed by maintenance gefitinib) is preferable to a “gamble” with gefitinib induction. Gefitinib may be reapproved in Canada, possibly in the restricted context of *EGFR-*mutated cancers.

## 3. NEW DEVELOPMENTS IN THE FIRST-LINE SYSTEMIC TREATMENT OF A-NSCLC

Several recent, well-conducted randomized trials involving pemetrexed, bevacizumab, cetuximab, erlotinib, and gefitinib indicate that previous management must be reconsidered.

### 3.1 Pemetrexed

Pemetrexed–cisplatin was tested against gemcitabine–cisplatin in first-line a-nsclc 15. This trial (jmdb), at 1725 accrued patients, is the largest randomized controlled trial ever conducted in this disease. The primary goal was non-inferiority in os, which was achieved (hr: 0.94; 95% confidence interval: 0.84 to 1.05 in favour of pemetrexed–cisplatin). Because of the unusual targeted nature of pemetrexed, an analysis by histology was pre-specified. As in the preceding second-line trial (jmei ), a pronounced difference by histotype was evident for the pemetrexed arm (interaction *p* = 0.0011): the adenocarcinoma (hr: 0.84; *p* = 0.03) and large-cell patients (hr: 0.67; *p* = 0.03) did better on cisplatin–pemetrexed than on cisplatin–gemcitabine, both separately and when grouped together as “non-squamous” (hr: 0.81; *p* = 0.005). The opposite was true for the squamous patients, who benefited more on gemcitabine–cisplatin than on pemetrexed–cisplatin (hr: 1.23; *p* = 0.05). Furthermore, objective response rates and pfs results either were all significantly different or trended in the same direction. One other noteworthy finding was that, on pemetrexed–cisplatin, the non-squamous patients did better than the squamous patients did [median survival time (mst): 11.8 months vs. 9.4 months], but on gemcitabine–cisplatin, the two histologic categories experienced very similar survival (mst: 10.4 months vs. 10.8 months).

Moreover, in ex-smoking, current-smoking, and never-smoking adenocarcinoma patients, pemetrexed–cisplatin was significantly superior to gemcitabine–cisplatin (Eli Lilly and Company. Data on file), with an identical adjusted hr of 0.82. Notably, in the uncommon large-cell subgroup, pemetrexed–cisplatin showed a hr of 0.67, and an important survival prolongation from 6.7 months to 10.4 months.

Although it is true that no trial result is currently available comparing a pemetrexed–platinum doublet with a taxane–platinum doublet in non-squamous patients, such trials are underway. Nevertheless, it is highly unlikely that cisplatin–gemcitabine is inferior to a platinum–taxane combination in such patients: compare, for example, the control arms of avail (Avastin in Lung Cancer) 46 and ecog 4599 47 for orr and pfs, at 6.2 months and 20% versus 4.5 months and 15%. It is therefore reasonable to propose that cisplatin–pemetrexed be considered a new standard of first-line care in patients with non-squamous disease.

A recent randomized phase ii study indicated that, in patients previously treated with cisplatin-based therapy, the addition of carboplatin to pemetrexed in the second line substantially improved pfs (hr: 0.67; *p* = 0.005) and orr, but not survival. Patients with non-squamous disease did better, regardless of treatment arm 48. This manoeuvre should be considered, because toxicity was very low.

I will shortly discuss another randomized trial (jmen) in the first line, in which pemetrexed maintenance resulted in a markedly differential effect by histology, consistent with jmdb and jmei. It is therefore now credible that pemetrexed–cisplatin is the current best chemotherapy for adenocarcinoma and large-cell disease in the first line, and that pemetrexed is the best available chemotherapy in the second-line in these histologies. Whether pemetrexed can be used in the first and the second line is uncertain, although experience in other cancers suggests that patients that enjoy a good response in the first line, and a relatively long pfs, will often respond again to the same drug or drugs in the second line 49.

A small randomized trial has established that pemetrexed–carboplatin is even less toxic than gemcitabine–carboplatin, but in that trial (and unusually), no difference by histology was apparent, and women survived longer on pemetrexed–carboplatin (hr: 1.43; *p* = 0.022) 50.

### 3.2 Bevacizumab

Bevacizumab, an anti-vegf monoclonal antibody, has been added to first-line chemotherapy in two trials: ecog 4599 47 and avail 46. In both trials, bevacizumab monotherapy was continued as maintenance. Eligibility was strictly limited to patients with non-squamous histology, no brain metastases, no significant hemoptysis, and no current anticoagulant therapy. The ecog 4599 trial, using carboplatin and paclitaxel, showed a 2-month survival benefit (10.3 months vs. 12.3 months); but by subgroup, only men benefited. Women and men both enjoyed a substantial increase in orr and pfs. The adenocarcinoma patients experienced a large survival benefit [10.3 months vs. 14.2 months: Sandler A, Yi J, Hambleton J, Kolb MM, Johnson DH. Treatment outcomes by tumor histology in Eastern Cooperative Group (ecog) study E4599 of bevacizumab (bv) with paclitaxel/carboplatin (pc) for advanced non-small cell lung cancer (nsclc). Presented at the 2008 Chicago Multidisciplinary Symposium in Thoracic Oncology; November 13–15, 2008; Chicago]. The avail trial, using cisplatin–gemcitabine, failed to show a survival benefit; however, orr and pfs again showed an important advantage. The mst for the avail control arm was unusually good, at 13.1 months—perhaps because a relatively high number of patients received post-discontinuation therapy.

A major and consistent advantage of bevacizumab is an increased response rate and pfs, thus potentially improving symptom control and time without symptomatic deterioration 39 and also reducing the fraction of patients that progress during the first line, thus increasing by 5%–10% absolute the number suitable for maintenance. Bevacizumab is contraindicated in patients with squamous disease because of the risk of exsanguinating hemoptysis.

The conflicting survival data in the two major trials, ecog 4599 and avail, have led to controversy regarding the ultimate value of bevacizumab; however, in respect of pfs and orr, the benefits seem secure.

### 3.3 Cetuximab

Cetuximab is a monoclonal antibody directed against the extracellular domain of egfr. In a large trial of first-line cisplatin–vinorelbine with or without cetuximab, survival was increased (hr: 0.803; *p* = 0.003), but only in Caucasian patients. Cetuximab may have a role in squamous carcinoma (hr: 0.794; mst: 8.9 months vs. 10.2 months) and in patients in whom bevacizumab is contraindicated. Note that cetuximab was continued as maintenance after completion of the cisplatin–vinorelbine 51.

### 3.4 Gefitinib

Gefitinib is widely used in East Asian patients because of a higher incidence of *EGFR* mutation, both in neversmokers and in light ex-smokers (approximately 60% vs. 30% in Western countries) and even in heavier ex-smokers (approximately 30% vs. approximately 10% in Western countries) 52. As mentioned, in *EGFR*mutated Asian never-smokers or light ex-smokers, gefitinib had statistically superior pfs and orr as compared with carboplatin–paclitaxel, but in the approximately 40% wild-type *EGFR* patients, gefitinib was markedly inferior to chemotherapy 45, with a 1.1% orr and a very short pfs. This finding has led to the suggestion that in *EGFR-*unknown patients, chemotherapy should be used in the first line, followed immediately by egfr-tki maintenance.

In interest, in which gefitinib was non-inferior to docetaxel in the second line (and less toxic), biomarkers did not select for differential benefit. Notably, objective response in the *KRAS-*mutated patients on gefitinib was 0% (and only 3.5% in those on docetaxel) 44.

## 4. MAINTENANCE THERAPY

Semantically, some practitioners prefer to restrict the term “maintenance” to trials in which the same first-line chemotherapy is continued beyond the 4–6 cycles of induction, in whole or in part, and to use “early second line” to describe the strategy of switching to another drug after induction, but before relapse. My preference is to use “maintenance” to describe either strategy.

Older trials showed no survival advantage for maintenance chemotherapy 53–56, but five recent trials indicate that such therapy should now be used. Brodowicz *et al.* 57 randomized patients benefiting from 4 cycles of induction cisplatin–gemcitabine to more gemcitabine or to observation. The pfs was better (hr: 0.7; *p* < 0.001) in the treated group; and in patients with a good performance status, os was markedly better (8.3 months vs. 22.9 months; hr: 0.48; *p* value significant). Fidias *et al.* 58 tested, in patients benefiting from induction doublets (excluding docetaxel), whether maintenance docetaxel compared with delayed (that is, second-line) docetaxel (at relapse) would offer superior pfs. It did (hr: 0.71; *p* = 0.0001), and os trended positively as well (9.7 months vs. 12.3 months; *p* = 0.0853). The large jmen 59 study, similar in design, but comparing pemetrexed with placebo in maintenance, showed a superior pfs, but only in patients with non-squamous disease (1.84 months vs. 4.37 months; *p* < 0.00001). Note that the induction 4 cycles of platinum doublet chemotherapy did not include a pemetrexed option. Final survival data are markedly positive: 10.3 months versus 15.5 months measured from randomization (*p* = 0.002) and 11.5 months versus 16.8 months in the adenocarcinoma subgroup (*p* = 0.026). Note that the disease control rate continued to increase during maintenance pemetrexed, to 49.1% from 28.9% (*p* < 0.09) in the entire intent-to-treat population.

The jmen trial has been criticized because a relatively low proportion (19%) of the control arm received second-line pemetrexed. However, 29% received docetaxel, which although inferior to pemetrexed, is not so inferior as would account for much of the large os difference. Furthermore, approximately one third received an egfr-tki (on both arms). This survival result, at the cost of minimal increases in grades 3 and 4 neutropenia (3% vs. 0%) and fatigue (5% vs. 1%) in a large (*n* = 663) well-conducted randomized controlled trial, is very provocative. Note that the actual survival should be increased by the (at least) 3-month induction chemotherapy period; in the adenocarcinoma maintenance arm, the median survival should therefore be at least 19.8 months. However, this extension should be interpreted in the knowledge that some percentage (about 45% 58) of patients that start first-line therapy are not able to proceed to maintenance and probably experience a rather short survival. Thus, median os for adenocarcinoma patients as a whole, starting from day 1 of cycle 1, would not approach 19.8 months, but rather somewhat less. If the median survival of the immediately-progressing patients was about 6 months, the median os for a postulated group of adenocarcinoma patients would decline to about 15 months. Note that the ecog 4599 adenocarcinoma subgroup (more highly selected) on carboplatin–paclitaxel–bevacizumab achieved a median os of 14.2 months [Sandler A, Yi J, Hambleton J, Kolb MM, Johnson DH. Treatment outcomes by tumor histology in Eastern Cooperative Group (ecog) study E4599 of bevacizumab (bv) with paclitaxel/carboplatin (pc) for advanced non-small cell lung cancer (nsclc). Presented at the 2008 Chicago Multidisciplinary Symposium in Thoracic Oncology; November 13–15, 2008; Chicago].

The two trials saturn (Sequential Tarceva in Unresectable NSCLC) and atlas (Adjuvant Tamoxifen —Longer Against Shorter) are looking at maintenance erlotinib, atlas in the context of bevacizumab. Both have met their pfs endpoint. Of these two studies, saturn is of most immediate relevance 60. The hr for pfs is 0.71, but the median pfs difference is negligible (12.3 weeks vs. 11.1 weeks). However, the mean pfs difference (22.4 weeks vs. 16 weeks) appears more worthwhile, and the orr (5.4% up to 11.9%) and the disease control rate (50.8% up to 60.6%) are increased. It does seem clear that certain subgroups benefited disproportionately 61, especially women (hr: 0.56), people of Asian ethnicity (hr: 0.58), never-smokers (hr: 0.56), patients positive for *EGFR* mutation (hr: 0.10), those with *EGFR* positivity by fluorescence *in situ* hybridization [fish (hr:0.68)] or immunohistochemistry (hr: 0.69), and those with wild-type *KRAS* (hr: 0.70). Of course, many of those groups overlap; what is needed is a multivariate analysis to better define the beneficiaries. It will then be worth considering maintenance erlotinib in a selection of the foregoing subgroups, especially but perhaps not only *EGFR-*mutated cases. In the remaining subgroups, evidence that is more compelling (that is, final os data, due in 2010) would be needed to consider maintenance erlotinib. The atlas results were similar, except that biomarker data are still to be presented. Overall, mean pfs increased by 1 month [3.75 months to 4.76 months (hr:0.722; *p* = 0.0012)], with people of Asian ethnicity, women, and never-smokers benefiting disproportionately. Data concerning os are due next year 62.

## 5. ANCILLARY STRATEGIES

One meta-analysis supports the use of the anti-emetic NK1 antagonist aprepitant in cisplatin-based chemotherapy 63. Suggestive randomized data also support the use of zoledronic acid in patients with bone metastases, even indicating a survival benefit (hr: 0.65; *p* < 0.001) 64. Prophylactic cranial irradiation could benefit patients with a high risk of brain metastases 65, as occurs with extensive small-cell lung cancer 66. Prophylactic anticoagulation should be explored in a-nsclc, as in small-cell disease 67.

### 5.1 The Algorithm

**FIGURE 1:**
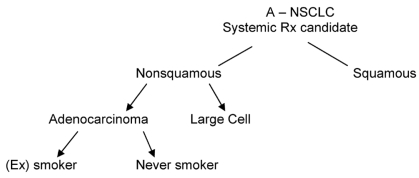
A suggested approach to the subgrouping of advanced non-small-cell lung cancer (a-nsclc) in the absence of biomarkers. Rx = prescription.

In the absence of biomarkers, I would propose the general scheme shown in Figure 1 for fit patients needing systemic therapy.

**FIGURE 2:**
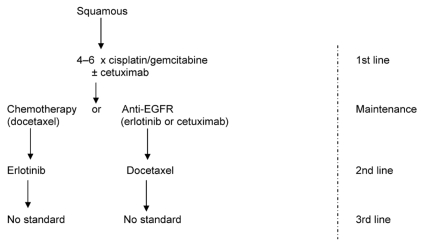
Suggested management of squamous cell carcinoma of the lung. egfr = epidermal growth factor receptor.

Squamous carcinoma (Figure 2) should be managed using 4–6 cycles of a platinum doublet, particularly cisplatin–gemcitabine. Carboplatin is an alternative, and vinorelbine and paclitaxel are also acceptable. Bevacizumab must be avoided, but cetuximab could be added in the first line. Maintenance could consist of cetuximab or docetaxel or erlotinib; observation alone is likely inferior. The second line should involve a crossover. Note that gefitinib use in the second line after docetaxel might be justified, but is not approved; the question of whether the higher-dose philosophy of erlotinib confers an advantage with egfr overexpression is open.

Biomarkers are probably not mandated in squamous pathology, because mutated-type *EGFR* does not occur, mutated-type *ras* is rare, and egfr overexpression by immunohistochemistry occurs in most patients. The role of egfr fish positivity needs better definition by histologic subtype.

**FIGURE 3:**
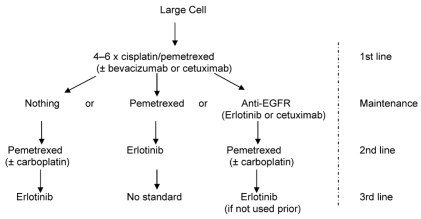
Suggested management of large-cell carcinoma of the lung. egfr = epidermal growth factor receptor.

Large-cell carcinoma patients (Figure 3) seem to benefit a great deal from pemetrexed-based chemotherapy, as in the first-line jmdb trial (hr:0.67; *p* = 0.03). In the jmen trial (pemetrexed maintenance), the number of large-cell cases was small, but pemetrexed should be considered if the first-line experience was good. Use of bevacizumab did not yield a survival benefit in the subgroup analysis of E4599, and its use might be justified only to enhance response in the first line. In the second line, erlotinib might be justifiable (unless the tumour is mutated-type *KRAS* or egfr fish-negative, or both), but data are scanty. Biomarker triage is not emphasized here, because information is scarce. The saturn study has not yet separately reported maintenance data for erlotinib in large-cell disease.

**FIGURE 4:**
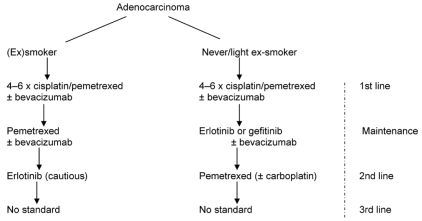
Suggested management of adenocarcinoma of the lung in the absence of biomarkers.

Adenocarcinoma management (Figure 4) is now complex and challenging. The schema here shows what would be reasonable in the absence of biomarkers. In this case, smoking status is the default triage. Cisplatin–pemetrexed is the established best first-line doublet, and use of pemetrexed in maintenance very likely prolongs survival; maintenance with an egfr-tki in never-smokers may be as good, especially in those of Asian ethnicity. Pemetrexed use in the first line and in maintenance, or alternatively in the first and second lines, is currently unsupported by data. If a choice has to be made, I would favour pemetrexed use for maintenance in ex- and current smokers (likely os benefit) and for the first line in never-smokers, followed by egfr-tki maintenance. Because approximately 40% of adenocarcinomas in ex- and current smokers show mutated-type *KRAS* (especially if poorly differentiated), erlotinib use here should be a cautious last resort. The never-smokers do not harbour classical activating mutated-type *KRAS.* But a small fraction do seem to harbour non-classical *KRAS* mutations of uncertain significance 68. The “right dose” of erlotinib in current smokers and recent ex-smokers may be 300 mg daily, because of enzyme induction 69, although this dose is not approved in Canada or anywhere else.

If biomarkers are available, then they should be the preferred triage over smoking status. Mutated-type *EGFR* has now emerged as the biomarker of main interest, because it so clearly delineates a distinct entity eminently treatable with first-line egfr-tki (especially gefitinib, were it to be approved), which is less toxic than erlotinib.

**FIGURE 5:**
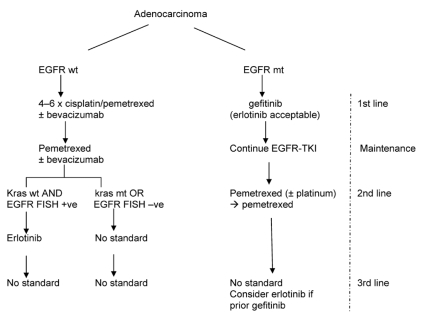
Suggested management of adenocarcinoma of the lung with biomarkers available. egfr = epidermal growth factor receptor; wt = wild type; mt = mutated type; tki = tyrosine kinase inhibitor; fish = fluorescence in situ hybridization; +ve = positive; –ve = negative.

In patients in Western countries with wild-type *EGFR,* the efficacy of egfr-tkis is uncertain; if these agents are effective, they are likely worthwhile mainly in patients with wild-type *KRAS* and fish-positive egfr (Figure 5).

### 5.2 Adenocarcinomas and Molecular Testing

Table II may be of assistance in respect of *KRAS* and *EGFR* mutational status in adenocarcinoma.

### 5.3 A Note About “Not Otherwise Specified”

Pathology reported as “NOS” (“not otherwise specified”) is not to be equated with large-cell disease. This result means either that the specimen was inadequate or that the cells are genuinely very undifferentiated, in which case mutated-type *EGFR* is very unlikely, and some additional guidance may be forthcoming from smoking status coupled with clinical features that commonly differentiate subtypes. For example, nonmetastatic hypercalcemia is probably exclusively found in squamous carcinomas. Table III, which shows stereotypes, may assist.

## 6. CONCLUSIONS

The algorithm provided here for a-nsclc patients with a good performance status may differ in some respects from current practice in Canada. In particular, I believe that maintenance chemotherapy, or biologic therapy, or both, should now be incorporated as a standard of care, although further optimization of drug and patient selection is a work in progress. For this reason, I do not believe that “all patients should get three lines of therapy.” Use of maintenance therapy may, in fact, remove a third-line option, but it guarantees that more people benefit. Furthermore, good evidence suggests that *KRAS-*mutant patients are generally not helped, and may be harmed, by egfr-tkis. In patients with *EGFR* mutations, gefitinib is probably equivalent to erlotinib, but in patients with egfr overexpression, the erlotinib dosing philosophy may hold an advantage over standard-dose gefitinib. I therefore believe that *KRAS* and *EGFR* testing should be available in adenocarcinoma patients, because “adenocarcinoma” is not a unitary entity. I suspect that the “right” dose of erlotinib in current and ex-smokers is 300 mg. I believe that the data with cisplatin–pemetrexed are good enough to justify first-line use both in adenocarcinoma and in large-cell disease. Bevacizumab should be considered to improve the depth of response, especially in patients with bulky or aggressive non-squamous disease. The role of bevacizumab in maintenance is debatable if chemotherapy or egfr-tki maintenance is to be used, but that agent should be considered. In patients with non-squamous disease, bevacizumab with carboplatin–paclitaxel may prolong survival, but cisplatin–pemetrexed may achieve the same survival even without bevacizumab. Cetuximab is a valid consideration in squamous carcinoma (and in other histologies if bevacizumab is contraindicated), but these two antibodies should not be used together. Never-smokers or light ex-smokers should receive cisplatin–pemetrexed followed immediately by maintenance with an egfr-tki; if they are known to have mutated-type *EGFR,* a first-line egfr-tki (either one) is acceptable, possibly with bevacizumab. Zoledronic acid use should be considered for patients at risk of a (or another) skeletal-related event, and prophylactic cranial irradiation might be considered once the risk factors for brain metastases are better understood. Prophylactic anticoagulation should be considered in high-risk patients. Palliative radiotherapy, and even surgery, should be considered for focal problems. Aprepitant improves the rate of control of cisplatin-induced nausea and vomiting.

If the foregoing measures are judiciously applied, the median survival for all types patients with a good performance status may exceed 1 year. Provincial provider agencies should carefully and responsibly consider the cost-effectiveness of these measures.

## Figures and Tables

**TABLE I:** Summary of grades 3 and 4 chemotherapy toxicity from selected large trials

*Reference*	*Regimen*	*Dose (mg/m^2^)*	*Schedule*	*Grade 3/4 toxicities (% of patients)*					
				*Febrile neutropenia*	*Anemia*	*Nausea/vomiting*	*Peripheral neuropathy*	*Platelets*	*Renal*
Kosmidis *et al.,* 2002^10^	Carboplatin, paclitaxel	auc 6 200 (over 3 hours)	Day 1, day 1, every 3 weeks	1	5	4	8	2	0
	Gemcitabine, paclitaxel	1000 200 (over 3 hours)	Days 1, 8; day 1; every 3 weeks	2	2	7	6	1	<1
	Carboplatin, paclitaxel, bevacizumab			5.2	0	nr	nr	1.6	nr
Schiller *et al.,* 2002^11^	Cisplatin, paclitaxel	75 135 (over 24 hours)	Day 2, day 1, every 3 weeks	16	13	25/24	5	6	3^a^
	Cisplatin, docetaxel	75 75	Day 1, day 1, every 3 weeks	11	15	14/21	5	3	3^a^
	Cisplatin, gemcitabine	100 100	Day 1; days 1, 8, 15; every 4 weeks	4^b^	28^b^	37/35	9	50^b^	9^a^,^b^
	Carboplatin, paclitaxel	auc 6 225 (over 3 hours)	Day 1, day 1	4^b^	10	9^b^/8^b^	10	10	1^a^
Fossella *et al.,* 2003^12^	Cisplatin, vinorelbine	100 25	Day 1; days 1, 8, 15, 22; every 4 weeks	5	24	16/16	4 (sensory)	4	nr
	Cisplatin, docetaxel	75 75	Day 1, day 1, every 3 weeks	5	7^c^	10/8^c^	4 (sensory)	3	nr
	Carboplatin, docetaxel	auc 6 100	Days 1, 8; day 8	4	10^c^	6/4^c^	1 (sensory)	7	nr
Georgoulias *et al.,* 2005^13^	Cisplatin, vinorelbine	80 30	Day 8; days 1, 8	nr	6	15^d^	nr	6	3
	Gemcitabine, docetaxel	1000 100	Days 1, 8; day 8	nr	2	2^d^	nr	4	0
Grønberg *et al.,* 2007^14^	Carboplatin, pemetrexed	auc 5 500	Day 1, day 1	nr	12	nr	nr	24	nr
	Carboplatin, gemcitabine	auc 5 1000	Day 1; days 1, 8; every 21 days	nr	13	nr	nr	54	nr
Scagliotti *et al.,* 2008^15^	Cisplatin, pemetrexed	75 500	Day 1, day 1	1.3	5.6	7.2/6.1	nr	4.1	nr

a Grades 3, 4, and 5 renal toxicity.

b Toxicity was significantly different from that for cisplatin paclitaxel (*p* < 0.05).

c Toxicity was significantly different from that for cisplatin vincristine (*p* < 0.01).

d Toxicity was significantly different between gemcitabine docetaxel and cisplatin vincristine (*p* < 0.001).

nr= not reported; auc = area under the curve.

**TABLE II:** Adenocarcinomas and molecular testing

*ras*	*EGFR*	*Smoking status*	*Histologic subtype*	*Therapeutic implication*
mt	wt	(Ex-)smokers: adenocarcinomas almost exclusively; never adenocarcinomas in a non-smoker^a^. About 40%–50% of smoking adenocarcinomas are *KRAS* mt in Western countries.	Mucinous bronchioloalveolar carcinoma (bac) strongly associated with classical *KRAS* mutations, but can occur in all adenocarcinoma subtypes; however, rarely in non-mucinous bac.	May suffer tumour acceleration (active harm) on epidermal growth factor receptor (egfr)–tyrosine kinase inhibitor (tki)? Chemotherapy probably effective in advanced setting.
wt	mt	Occurs in ±30% of never- or light ex-smokers in Western countries. Occasionally seen (approximately 10%–12%) in (ex-)smokers in Western countries.	Non-mucinous bac (±50%) and papillary adenocarcinoma (35%) associated with *EGFR* mt.	Very likely to benefit substantially from egfr-tki. Chemotherapy also quite effective, maybe more so than in *EGFR* wt.
wt	wt	(Ex-)smoker: a slight preponderance (50%–60%) of smoker adenocarcinomas in Western countries; virtually all squamous cell carcinomas.	Any histotype, squamous or non-squamous; but if bac present, likely non-mucinous (approximately 50% *EGFR* wt)	Chemotherapy is the main option. Benefit from tki is unknown; may depend on overexpression of egfr (by fluorescence *in situ* hybridization)
wt	wt	Never-smoker: about 70% of never- or light ex- smokers in Western countries.	bac, if present, is non-mucinous.	Not helped by gefitinib [probably as good as placebo (? passive harm)]; chemotherapy somewhat effective. Effect of erlotinib unknown.

a A minority of never-smokers have non-classical (transition) *KRAS* mt of unknown significance.

mt = mutated type; wt = wild type.

**TABLE III:** Stereotypes in lung cancer^a^

	*Squamous*	*Adenocarcinoma*		*Large-cell*
		*Current or ex-smoker*	*Never-smoker*	
Pathology	Typical	bac component; may be mucinous; ttf-1 helpful	Any bac component will be non-mucinous; ttf-1 helpful	Heterogonous; may have neuroendocrine features
Demography	Often an older male, heavy (ex-) smoker	Male > female	Female > male; any age; common in East Asian individuals	Often an older male, heavy smoker
Clinical	Often proximal primary, may cavitate, paraneoplastic hypercalcemia, clubbing prominent, hemoptysis risk high	Primary may be more distal; can have clubbing; brain metastases, especially if female or elevated ldh	Primary may be distal, can be multifocal; tendency to brain metastases; do not have clubbing	Aggressive cancer; may metastasize to brain
Molecular	*ras* mutated type rare; *EGFR* mutated type nonexistent (?); egfr overexpression (by ihc) common; egfr fish+ ±25%; higher ts levels	*ras* mutated type approximately 45% in North America (approximately 17% in Asia), especially poorly differentiated; *EGFR* mutated type occurs, but <10% in North America (approximately 30% in Asia); lower ts levels; incidence of egfr fish+ (approximately 25% ?)	*EGFR* mutated type occurs in approximately 60% Asian individuals, and approximately 30% Caucasian individuals; especially well differentiated; activating codon 12/13; *KRAS* mutations do not occur; lower ts levels; non-classical *ras* mutations may occur; significance (?)	*ras* mutation incidence unclear, may be rare; no *EGFR* mutations; TS levels (low, unless neuroendocrine subtype); egfr fish (?)

a The relationships between clinical stereotypes, histology, and biomarkers are generally tentative. This table should be used with caution; it is intended to provide clues when definitive histologic or biomarker data (or both) are unavoidably lacking.

bac = bronchioloalveolar carcinoma; ttf-1 = thyroid transcription factor-1; ldh = lactate dehydrogenase; ihc = immunohistochemistry; fish = fluorescence *in situ* hybridization; ts = thymidylate synthase.
